# Design of a Modular Wall-Climbing Robot with Multi-Plane Transition and Cleaning Capabilities

**DOI:** 10.3390/biomimetics10070450

**Published:** 2025-07-08

**Authors:** Boyu Wang, Weijian Zhang, Jianghan Luo, Qingsong Xu

**Affiliations:** Department of Electromechanical Engineering, Faculty of Science and Technology, University of Macau, Avenida da Universidade, Taipa, Macau, China; mc35203@um.edu.mo (B.W.); yc27452@um.edu.mo (W.Z.); mc35202@um.edu.mo (J.L.)

**Keywords:** wall-climbing robots, modular robots, wall transition, mechanical design

## Abstract

This paper presents the design and development of a new modular wall-climbing robot—Modular Wall Climbing-1 (MC-1)—for solving the problem of autonomous wall switching observed in wall-climbing robots. Each modular robot is capable of independently adhering to vertical surfaces and maneuvering, making it a fully autonomous robotic system. Multiple modules of MC-1 are connected by an electromagnet-based magnetic attachment method, and wall transitions are achieved using a servo motor mechanism. Moreover, an ultrasonic sensor is employed to measure the unknown wall-inclination angle. Mechanical analysis is conducted for MC-1 at rest individually and in combination to determine the required suction force. Experimental investigations are performed to assess the robot’s crawling ability, loading capacity, and wall-transition performance. The results demonstrate that the MC-1 robot is capable of multi-angle wall transitions for executing multiple tasks. It provides a new approach for wall-climbing robots to collaborate during wall transitions through a quick attachment-and-disassembly device and an efficient wall detection method.

## 1. Introduction

Various types of vertical surfaces, such as building facades, window panes, and swimming pool walls, require regular cleaning due to the accumulation of dust and pollutants. Ensuring cleanliness, hygiene, and the overall aesthetic appeal of a wall surfaces is crucial. To meet the growing demand for cleanliness and hygiene, traditional manual cleaning methods are not only inefficient but also pose safety risks [1,2]. Typically, human labor is employed to clean different wall surfaces using tools such as pressure washers, rags, and brooms. Such a labor-intensive process is strenuous and repetitive. Furthermore, the wall structures are diverse with different angles and designs, which require the frequent repositioning of cleaning equipment. In some cases (particularly in narrow spaces), it is difficult for a human worker to perform effective cleaning operations, signifying the need for miniaturized cleaning robots. The inconveniences associated with the current cleaning methods underscore the necessity of designing a new robot capable of cleaning walls with various inclination angles.

Due to the varying working environments and task requirements, the walking mechanisms of wall-climbing robots differ greatly [3]. To date, the primary locomotion mechanisms of wall-climbing robots include wheels, crawlers, and legs [4,5]. In particular, wall-climbing robots with wheeled mechanisms are common due to their flexibility in turning and high-speed mobility. These wheeled robots can adopt various types of wheels, such as magnetic wheels [6,7] and rubber wheels [8], with different styles for specific operational scenarios. Crawler-type wall-climbing robots are powered by two motors positioned on either side of the tracks, enabling movement via differential steering. The larger contact area between the tracks and wall leads to increased friction, which enhances the stability of the robot’s movement on the wall. Additionally, the tracks provide a greater driving force on rough surfaces, and their unique mechanical design creates a vacuum cavity in conjunction with a fan, further strengthening the robot’s adhesion capabilities [9]. Crawler movement can also be combined with magnetic adhesion for robots equipped with permanent magnet attachment mechanisms, allowing them to climb on iron tanks with magnetic surfaces [10]. Wall-climbing robots with legged mobility are typically used in scenarios where large obstacles need to be overcome. The flexibility of leg movements allows these robots to seamlessly transition between walking and climbing modes, enabling them to move easily across walls [11]. Even simple bipedal robots can overcome large obstacles and carry smaller sub-robots to perform collaborative tasks [12].

The design of the adhesion device is the core technology needed for the wall-climbing robot to realize movement on walls via negative-pressure adhesion, magnetic adhesion, bionic adhesion, and electrostatic adhesion [4,5]. Negative pressure generates a thrust by creating a low-pressure region in a specific area, allowing relatively high-pressure atmospheric flow to enter the low-pressure region. The commonly used methods involve blades that create a rotating flow within the suction cup’s housing and suction cups that generate negative pressure for adhesion [13,14]. Magnet adhesion robots can be classified into two categories: electromagnet and permanent magnet adhesion. In both types, the adhesion to the wall of a conductor is facilitated by magnetism [15,16]. Alternatively, bio-adsorption is achieved by mimicking natural structures (such as gecko foot pads, utilizing Van der Waals forces) or by replicating the hooks found on insect legs and feet to enable adsorption on rough surfaces [17,18]. Electrostatic adhesion relies on electrostatic induction to achieve adhesion, allowing the robot to perform movement (such as forward and backward motion, as well as turning) on flat surfaces [19]. Additionally, thrust adhesion involves pressing the robot against wall using reverse thrust generated by a fan, with the robot’s movement being controlled by such a force [20].

Recently, wall-climbing robot technology has been advanced greatly, leading to the emergence of a wide variety of wall-climbing robots for different wall-related tasks. To improve efficiency, expand the range of applications, increase the number of working scenarios, and reduce the need for human intervention in maintenance, many robots have adopted modular designs with enhanced capabilities for wall transitions. For instance, the R-Track robot features a modular design, for which its modules can be connected and separated via connectors to adapt to different climbing environments [15]. R-Track employs a magnetic adhesion crawler movement method. However, such an approach is limited to magnetically conductive materials. Another example is a modular bionic climbing robot that uses articulated and adhesion modules to climb on vertical glass walls [21]. Vega-Heredia et al. designed a modular glass curtain wall-cleaning robot, “Mantis”, consisting of three modules. This robot can traverse metal and window frames [22]. While “Mantis” is capable of translating between parallel planes, it cannot transition between angled surfaces. The robot’s obstacle-crossing ability is constrained by the spacing between its modules. Furthermore, some modular, tracked climbing robots adopt passive flexible joints and dry elastomer adhesion techniques [23]. Such robots can transition between smooth and rough surfaces. However, due to the limitations of dry elastomer adhesion, they can only operate on dry surfaces and carry lower payloads. The ROMERIN modular robot combines suction-cup negative-pressure adhesion with legged locomotion, offering high maneuverability and loading capacities. However, it is larger in footprint and heavier compared to other designs [24].

This paper presents the design and development of a novel modular wall-cleaning robot with multi-plane transition capabilities, named MC-1. Vacuum suction is implemented for adhering the robot to wall, ensuring its attachment on multiple surfaces. The main contributions of this paper are summarized as follows:

- A modular design is realized with multiple modules connected by servo motors and magnets. Each module has the same structure and composition, and they can be connected in series with each other and realize mutual lifting. The modules can be quickly assembled and disassembled, enhancing flexibility and simplifying maintenance work.
- The combination of servo motors and ultrasonic sensors is introduced for wall-angle measurement, allowing the robot to autonomously adapt to different wall planes. This new detection method is computationally fast and has a simple detection structure, which makes it suitable for low-cost confined-space use.
- The mechanical theory analysis of the robot was carried out under a variety of attachment conditions to calculate the required minimum suction force, which is verified by experiments.

The remaining parts of this paper are organized as follows. Section 2 details the mechanism design and system configuration of MC-1. Section 3 discusses the force analysis and the methodology for wall-angle measurement in the context of wall-to-wall transitions. Section 4 presents the simulation study and experimental results. Finally, Section 5 concludes this paper.

## 2. Design of the MC-1 Robot

### 2.1. Components and Electrical System Architecture

**Figure 1:**
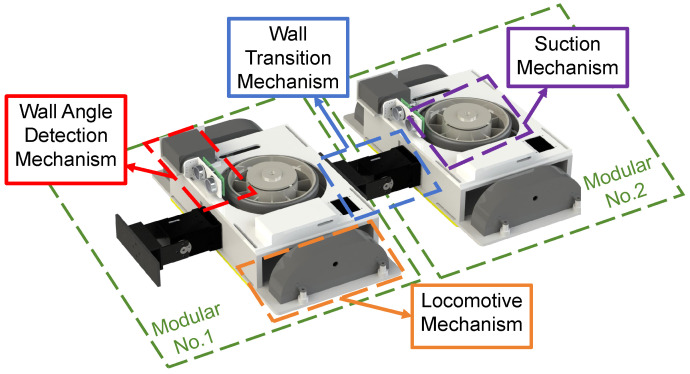
Component of MC-1. The MC-1 robot is a modularly designed wall-climbing robot with the same structure for each module, including a wall-angle detection mechanism, wall-transition mechanism, suction mechanism, and locomotive mechanism.

In this work, MC-1 is designed to climb on and clean the inner walls of a pool while switching between multiple walls as a case study. As shown in Figure 1, MC-1 consists of two modular robots. Each module comprises four main components: a suction mechanism, a locomotive mechanism, a wall-angle detection mechanism, and a wall-transition mechanism. The cavity at the bottom of the blower and the chassis is primarily responsible for providing the adhesion force, which is the main element of the adhesion mechanism. The locomotion mechanism is constructed by a rubber track and an integrated drive motor.

The wall-transition mechanism includes a servo motor and an electromagnetic attachment module. The servo motors (model: SM45BL, from Shenzhen Feetech RC Model Co., Ltd., Shenzhen, China) mounted with a DT4 steel plate and electromagnet are magnetically connected to facilitate the wall transition for the MC-1 robot modules. The wall-angle detection mechanism includes servo motors and an ultrasonic sensor. The servo motor (model: MG945, from Tower Pro Pte Ltd., Singapore) and an ultrasonic sensor (model: US-015, from Manorshi Electronics Co., Ltd., Changzhou, China) are used to detect the wall angle. The cleaning device mainly consists of a cleaning cloth installed at the bottom of the robot.

**Figure 2:**
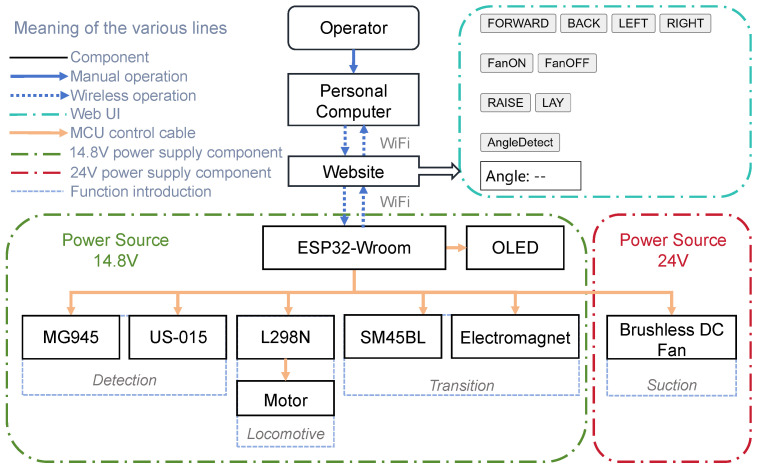
Electrical system architecture diagram of MC-1. The main functions of MC-1 can be controlled by the operator via a web interface. The ESP32 wireless module on MC-1 communicates wirelessly with the host computer and controls the components through cables based on the commands received from the host computer.

The system framework of the MC-1 robot is illustrated in Figure 2. It consists of a wireless module (model: ESP32-WROOM, from Espressif Systems (Shanghai) Co., Ltd., China), which communicates with the computer via its built-in Wi-Fi module. The Wi-Fi base station assigns a unique IP address to each wireless module (ESP32), creating a web page through which the user can control the MC-1 robot by tapping and long-pressing buttons. The user interface (UI) is displayed on the right side of the web page. The ESP32-WROOM module controls a motor driver (model: L298N, from STMicroelectronics N.V., Geneva, Switzerland) to manage the robot’s movement and operates the MG945 servo motor and US-015 ultrasonic sensor to measure the wall angle. Additionally, it controls the SM45BL servo motor and solenoid, which are used to interact with other modules of the robot and perform the wall-switching action. The OLED screen connected to the ESP32 displays the current IP address of the MC-1 module, simplifying user operations. Such a feature allows the user to connect to the robot by directly entering the IP address into the web page, eliminating the need to obtain the IP address through a bus.

### 2.2. Main Mechanisms of MC-1

**Figure 3:**
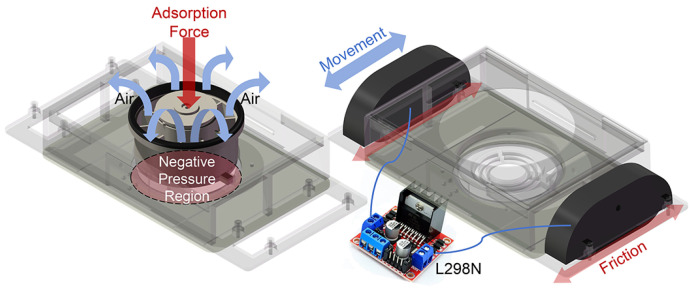
Suction and locomotive mechanism. The fan creates a negative-pressure zone between the bottom of the robot’s MC-1 chassis and the wall surface by drawing air from the surrounding environment, allowing the robot to adhere to the wall. Movement of MC-1 is achieved by the frictional sliding of the tracks against the wall.

As shown in Figure 3, the suction mechanism utilizes a DC brushless fan (diameter: 94 m; model: 35N709L070, from Nidec Precision Corp., Tokyo, Japan) with a maximum speed of 20,000 rpm. The mobile unit employs a motor driver module (L298N) to control two motors (XR385-19112), which drive rubber tracks to propel the MC-1 module. The robot’s movement is controlled through forward and reverse rotation, enabling it to move forward and backward and steer. Compared to wheel-based driving systems, rubber tracks provide greater friction, thus offering more driving force and ensuring the smoother movement of the robot.

**Figure 4:**
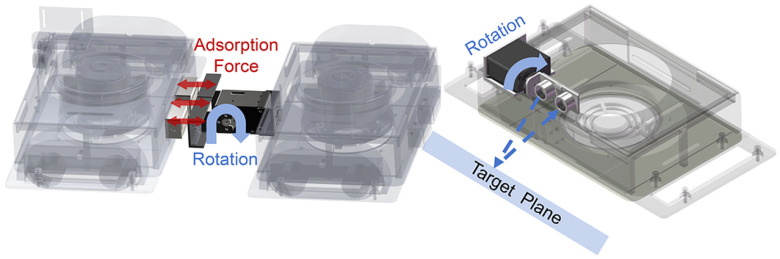
Detection and transition mechanism. As the servo motor rotates, the ultrasonic sensor measures the distance, which is then used to calculate the angle. The electromagnets can generate an attraction force of 200 N in attachment direction. After the two modules are attached electromagnetically, the servo motor rotates to complete the wall transition.

Figure 4 illustrates the wall-angle detection mechanism and wall-transition mechanism. The wall-transition mechanism consists of three components: a magnetic connection system, a wall-angle detection device, and a servo motor for wall adaption. The magnet connection system includes two electromagnets (dimension: 40 mm × 20 mm × 20 mm) and an iron plate connector made of DT4 material. The electromagnets are magnetized and demagnetized rapidly to attach to and release from the stainless steel connector in the case of power failure. The wall-angle detection device consists of a servo motor (MG945) and an ultrasonic sensor (US-015).

## 3. Working Principle and Analysis of MC-1 Robot

**Figure 5:**
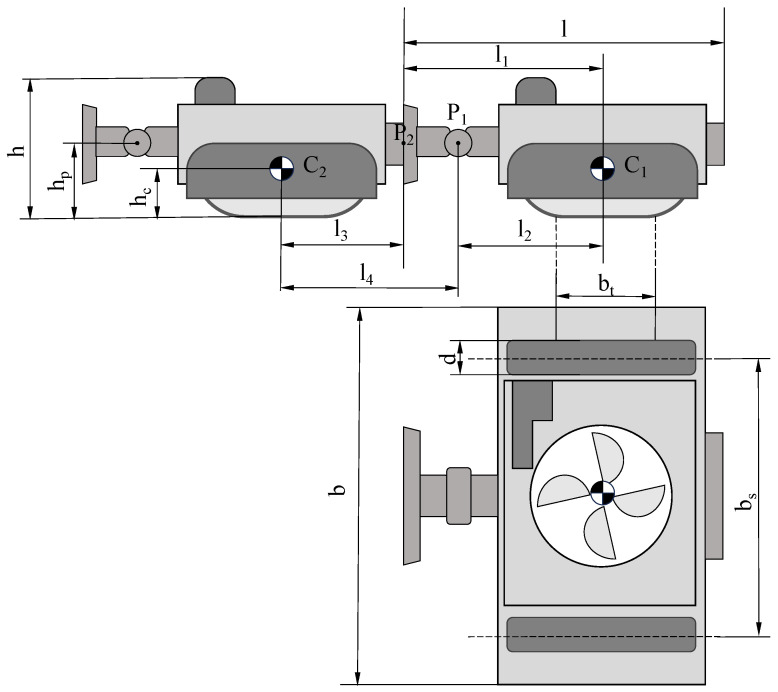
Simplified diagram of the module of MC-1.

The structure of the MC-1 robot is simplified, as shown in Figure 5. Point $C_{i}$ represents the position of the center of gravity of the *i*-th modular robot, $P_{1}$ is the center of the rotation of the wall converter in the first module, and $P_{2}$ is the center of the tail electromagnet surface on the second module. The length of a single modular robot is denoted as *l*. The center of gravity of the module is essentially coaxial with the central axis of the fan, which is achieved by distributing the front and rear masses and arranging the internal electronics accordingly. The distance from the front plane of the module to the center of gravity is $l_{1}$, the distance from the center of rotation of the wall converter to the center of gravity is $l_{2}$, the distance from the surface of the electromagnet at the tail of the module to the center of gravity is $l_{3}$, and the distance from the center of gravity of the second module to the center of rotation of the servo motor in the first module is $l_{4}$. The height of the individual modular robot is *h*, and the height of the center of gravity is $h_{c}$. The width of the individual modular robot is *b*, and the width of the center line of both tracks is $b_{t}$. The width of the track is *d*, and the distance from the center of rotation of the wall converter to the plane of the track is $h_{p}$. The specific dimensional parameters of each part are tabulated in Table 1.

### 3.1. Static Mechanical Analysis

As a case study, the application scenario of the MC-1 robot is assigned as the inner wall of a water pool. Generally, the inclination angle of the inner wall ranges from 0° to 90°. Thus, we focus on the force analysis of the robot in this angle range. The force of MC-1 is mainly divided into the following categories: the force of stable adhesion on the vertical wall, the force of plane-to-vertical wall conversion, and the force of the transition between two vertical walls.

**Figure 6:**
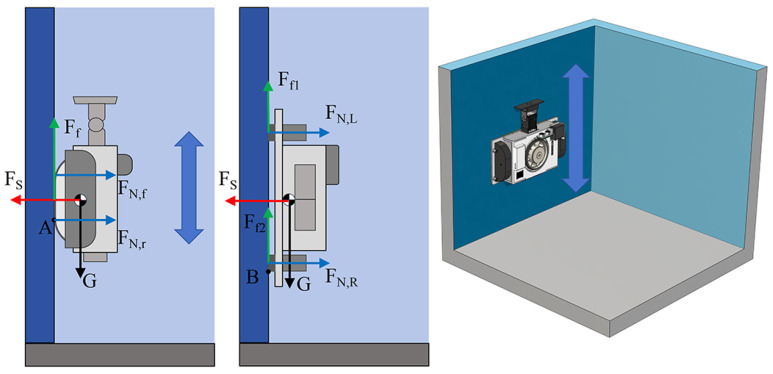
Static analysis of a single MC-1 module adhering on the wall.

As shown in Figure 6, the figure demonstrates the forces on MC-1 when crawling on a vertical wall, including the robot’s own gravitational force *G*, the suction force $F_{S}$ provided by the fan, and the support force $F_{N}$ exerted by the wall on the robot. $F_{N,f}$ and $F_{N,r}$, respectively, denote the wall support forces on the front and rear track wheels.

When the robot is at rest on the wall or in the state of traveling at a constant speed, there should be sufficient static friction between its tracks, cleaning cloth, and the wall. In the following, the downward sliding of the MC-1 robot on the vertical wall is analyzed. In the critical condition of downward slips, the robot should satisfy the following equations:

(1) $$\begin{array}{c} \left\{\begin{array}{cc} F_{f}-G & ⩾\;\;0\\ F_{f}-\mu \left(F_{N,f}+F_{N,r}\right) & =\;\;0\\ F_{S}-\left(F_{N,f}+F_{N,r}\right) & =\;\;0 \end{array}\right. \end{array}$$

where μ (magnitude: 0.62) is the friction coefficient between the robot track and the wall. G = mg, m is the weight of a single MC-1 module (m = 1.35 kg), and *g* is the acceleration of gravity (g = 9.8 m^2^/s). It is derived that the suction force $F_{S}$ should satisfy the following condition:

(2) $$\begin{array}{c} F_{S}\geq \frac{G}{\mu } \end{array}$$

Then, $F_{S}\geq 21.77$ N can be calculated. In addition to considering the downward sliding of the robot, when MC-1 moves on the wall, its negative-pressure suction force generates a moment greater than the overturning moment due to gravity. Therefore, the forces in both longitudinal and lateral overturning states should also be considered.

First, the longitudinal movement of MC-1 may result in tipping around point *A*. To ensure stable motion, the robot should meet the following conditions:

(3) $$\begin{array}{c} \left\{\begin{array}{cc} M_{A1} & \geq \;\;M_{A2}\\ M_{A1} & =\;\;F_{S}\frac{1}{2}b_{t}\\ M_{A2} & =\;\;F_{N,f}b_{t}+Gh_{c}\\ F_{N,r} & =\;\;F_{S} \end{array}\right. \end{array}$$

where $M_{A1}$ is the suction moment, $M_{A2}$ is the overturning moment, $F_{N,f}$ is the support force on the upper side of the track, and $F_{N,r}$ is the support force on the lower side of the track. At the critical point of imminent overturning, $F_{N,f}$ = 0. Then, it is found that the suction force at this moment should satisfy the following:

(4) $$\begin{array}{c} F_{S}\geq 2G\frac{h_{c}}{b_{t}} \end{array}$$

When MC-1 travels laterally, there is a situation where it rolls over sideways around point *B* under the effect of gravitational moment. The robot should satisfy the following equations to ensure stability:

(5) $$\begin{array}{c} \left\{\begin{array}{cc} M_{B1} & \geq \;\;M_{B2}\\ M_{B1} & =\;\;\frac{1}{2}F_{S}\left(b_{s}+d\right)\\ M_{B2} & =\;\;F_{N,L}\left(b_{s}+\frac{1}{2}d\right)+\frac{1}{2}F_{N,R}d+Gh_{c}\\ F_{P} & =\;\;F_{N,R} \end{array}\right. \end{array}$$

where $M_{B1}$ is the suction moment, $M_{B2}$ is the overturning moment, $F_{N,L}$ is the support force on the upper side track, $F_{N,R}$ is the support force on the lower side track, and $F_{N,L}=0$ at the overturning critical point.

After simplification, the suction force should satisfy the following:

(6) $$\begin{array}{c} F_{S}\geq 2G\frac{h_{c}}{b_{S}} \end{array}$$

**Figure 7:**
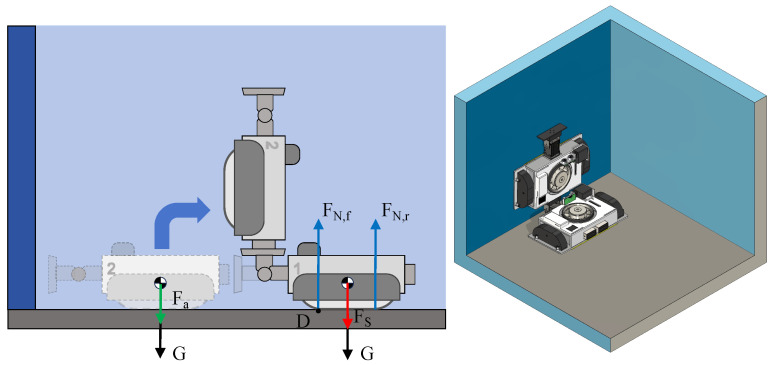
Static analysis of MC-1 modules during wall transition.

The vertical wall transition is shown in Figure 7. When MC-1 (with two modules) is performing a wall transition, sufficient suction force is required to ensure that the whole process proceeds smoothly and to prevent the robot from tilting around point *D*. The suction force is also required to prevent the robot from tipping over. In addition, when the servo motor lifts the front module, the angular acceleration of the servo motor’s rotation generates a force $F_{a}=\frac{I\alpha }{l-\frac{1}{2}b_{t}}=0.04$ N, and the front side module is simplified as a rod, where *I* is the rotational inertia, i.e., $I=\frac{m{\left(l-\frac{1}{2}b_{t}\right)}^{2}}{3}$, and α is the angular acceleration, i.e., $\alpha =\frac{\pi }{6}$ rad/s^2^. When the robot at the rear module begins to lift the front module, the maximum suction force $F_{S}$ is required at this moment. Thus, the following equations can be obtained:

(7) $$\begin{array}{c} \left\{\begin{array}{cc} M_{D1} & \geq \;\;M_{D2}\\ M_{D1} & =\;\;\frac{1}{2}\left(F_{S}+G\right)b_{t}\\ M_{D2} & =\;\;\left(G+F_{a}\right)\left(l-\frac{1}{2}b_{t}\right)+F_{N,r}b_{t}\\ F_{N,f} & =\;\;F_{S}+2G \end{array}\right. \end{array}$$

where $M_{D1}$ is the suction moment and $M_{D2}$ is the overturning moment. At the critical point of impending overturning, the rear side is $F_{N,r}=0$. Hence, it can be derived that the suction force $F_{S}$ should satisfy the following:

(8) $$\begin{array}{c} F_{S}\geq \frac{2(G+F_{a})l}{b_{t}}-2G-F_{a} \end{array}$$

Combining the force analyses of the above cases, it is obtained that the suction force $F_{S}$ should satisfy the following condition:

(9) $$\begin{array}{c} F_{S}\geq \max\left\{\frac{G}{\mu },\frac{2Gh_{c}}{b_{t}},\frac{2Gh_{c}}{b_{s}},\frac{2(G+F_{a})l}{b_{t}}-2G-F_{a}\right\} \end{array}$$

The calculation shows that $F_{S}$ should be not less than 27.02 N to ensure that the MC-1 robot remains stable in operation.

### 3.2. FEA Simulation Study

**Figure 8:**
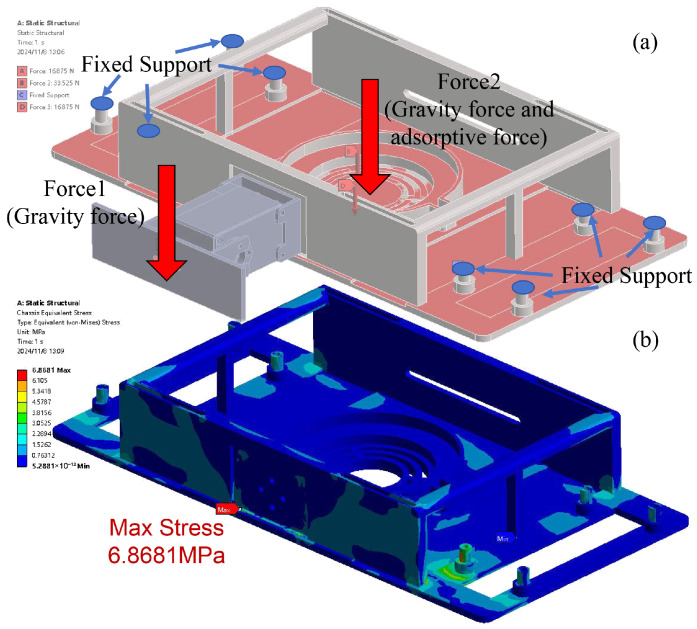
FEAsimulation result of chassis. (**a**) Setting of chassis loads and constraints; (**b**) distribution of equivalent stress of the chassis.

As the chassis of the MC-1 module is 3D-printed from Polylactic Acid (PLA) material, it is mechanically weaker than the metal materials to which it is attached. To ensure that the main structure of the robot will not be damaged during operation, a finite element analysis (FEA) of the chassis is conducted to assist in the chassis’s design. The initial state of the wall transition between two MC-1 robots (Figure 7) was chosen for the FEA study, where Module No. 1’s front attachment is subjected to Module No. 2’s gravity *G*, and the chassis is subjected to gravity and suction forces in the downward direction, i.e., $F_{S}+G$ = 40.32 N. To account for real-world conditions, including servo motor acceleration, and to prevent slippage, the loads are multiplied by a safety factor (S=1.25). It is assumed that the tracks do not slip, i.e., the constraints are that the fixed tracks do not move upon connection with the chassis. The simulation results are shown in Figure 8.

According to the stress distribution map, it is found that there are larger stresses at the connection between the servo motor and the bottom plate and at the connection between the track module and the bottom plate. Thus, these parts are thickened and rounded to improve structural strength and avoid stress concentration. The results of the FEA simulation study show that the maximum stress on the chassis of the MC-1 module is 6.8681 MPa, which is far lower than its tensile strength of 40 MPa and bending strength of 68 MPa. The results indicate that the structural material is sufficiently safe.

### 3.3. Detection of Wall Inclination Angle

**Figure 9:**
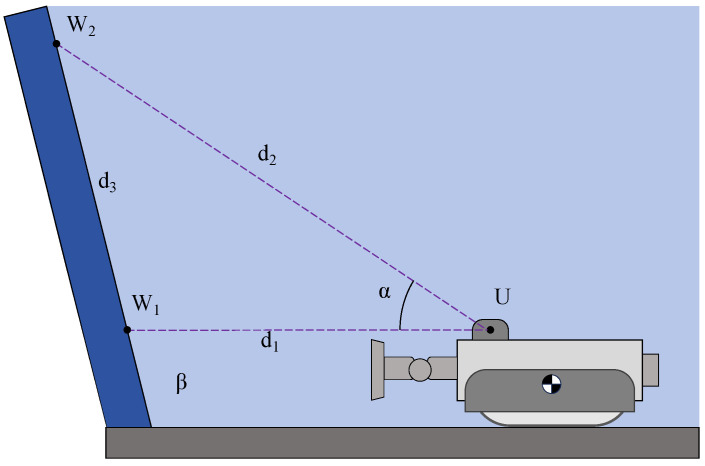
Principle of detecting the wall inclination angle using an ultrasonic sensor.

The MC-1 modular wall-cleaning robot is capable of detecting and navigating multi-angle surfaces. The wall-angle detection system primarily consists of a servo motor (MG945) and an ultrasonic sensor (US-015). Figure 9 shows the principle of the wall-angle measurement carried out by the robot. First, the robot finds the minimum distance value $d_{1}$ between the measuring center point of the ultrasonic sensor and the wall surface by swinging left and right. At this moment, the MC-1 robot is facing the wall surface. *U* is the measuring center point of the ultrasonic sensor, and $W_{1}$ is the closest point of the wall surface to the point *U*. Then, the robot can detect the angle of the wall surface by rotating the servo motor. After this, the servo motor’s rotation is set as the MG945 servo motor’s angle α, and it is measured again to obtain the distance $d_{2}$ from point *U* to a point $W_{2}$ on the wall surface. Assuming that the distance between point $W_{1}$ and point $W_{2}$ is $d_{3}$ and the angle between the wall surface and the bottom surface is β, the following equations can be generated:

(10) $$\begin{array}{c} \left\{\begin{array}{cc} \cos\alpha & =\frac{d_{1}^{2}+d_{2}^{2}-d_{3}^{2}}{2d_{1}d_{2}}\\ \sin\alpha & =\sqrt{1-{\left(\cos\alpha \right)}^{2}}\\ \frac{d_{3}}{\sin\alpha } & =\frac{d_{2}}{\sin\beta } \end{array}\right. \end{array}$$

## 4. Experimental Results

### 4.1. Testing Result of Suction Force

**Figure 10:**
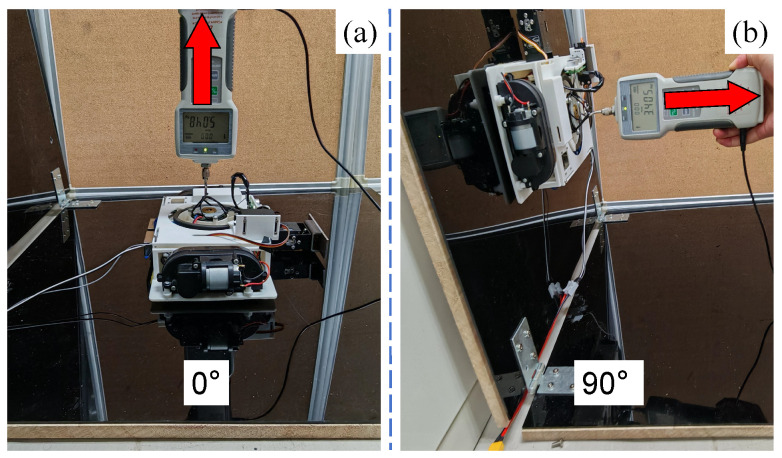
Suction force test of the MC-1 module. (**a**) shows the test performed by a dynamometer on a horizontal plane applying a tensile force to the module, the result of which needs to be subtracted from the module’s own gravitational force. (**b**) demonstrates a test on the suction force applied to the module on a vertical wall surface. The red arrows indicate the applied force directions.

The adhesion capacity is one of the most critical performance parameters for wall-climbing robots, as it determines whether the wall-climbing robot can complete basic movement. The minimum value of the suction force $F_{S}$ was calculated to satisfy various working conditions in the above section, as given in Equation (9), i.e., the suction force $F_{S}$ should not be less than 27.02 N. Therefore, the suction force of the MC-1 robot was tested. As shown in Figure 10, a single MC-1 module was placed on a vertical wall surface and a horizontal wall surface, and a dynamometer was placed at the center of gravity of the module after the MC-1 module had stabilized. A tensile force perpendicular to the wall surface was applied to the module and gradually increased until the robot was detached from the adhesion plane, and the value of the tensile force was recorded. The force perpendicular to the wall was subtracted from the gravitational force of the module *G*, and the experiment was repeated 10 times with the mean and standard deviation calculated.

As shown in Table 2, the minimum value of the suction force $F_{S}$ in both planes is greater than 27.02 N, which can satisfy the suction force requirement of the calculation.

The data in the table show that the value of the suction force $F_{S}$ is generally higher in the 0° horizontal wall plane than in the 90° vertical wall plane. It is presumed that the reason for this is that when the MC-1 module is placed horizontally, due to the additional effect of gravity, the vacuum cavity between the wall-crawling robot and the wall is more airtight, and thus, the value of the tensile force for the detachment of MC-1 from the wall is higher.

### 4.2. Testing Result of Loading Capacity

Loading capacity is a key indicator of the wall-climbing robot’s adhesion ability. It plays an important role in the integration and application of new components in future developments. In order to obtain the load capacity of the MC-1 robot, we tested the robot in both vertical and horizontal load directions by mounting a dynamometer on a wall within a range of 0–90° and in groups of 10° for a total of 10 sets of experiments. Each set of experiments is repeated for the same wall angle and in the same direction, and 10 sets of data are measured to take the mean and its standard deviation, which is used to estimate the load capacity of the robot in that direction.

**Figure 11:**
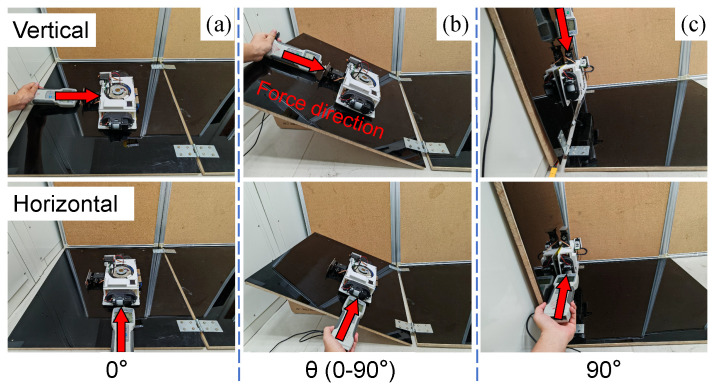
Loading test of the MC-1 module. (**a**–**c**) illustrate the location and direction of horizontal and vertical loads applied to MC-1 when adhered to different angular planes. The red arrows indicate the loading directions.

As shown in Figure 11, a dynamometer is placed along the plane at an inclined angle in the forward direction of the MC-1 robot, and after MC-1 is firmly attached, the robot is pressed, and the force is gradually increased; the minimal value of the force that causes the robot to move is recorded to obtain the vertical load data. Similarly, the force meter is placed horizontally perpendicular to the forward direction of MC-1 to measure the horizontal load data. In Figure 11a–c, the images on the upper row show vertical loads, and the images on the bottom row show horizontal loads. The specific data are tabulated in Table 3. The table lists the means and their standard deviations of the load values obtained from the 10 groups of tests. The coefficient of variation (CV) of the data (i.e., the ratio $\frac{\delta }{\mu }$ of the standard deviation to the mean) is low, which reveals that the consistency of the measurements is high.

**Figure 12:**
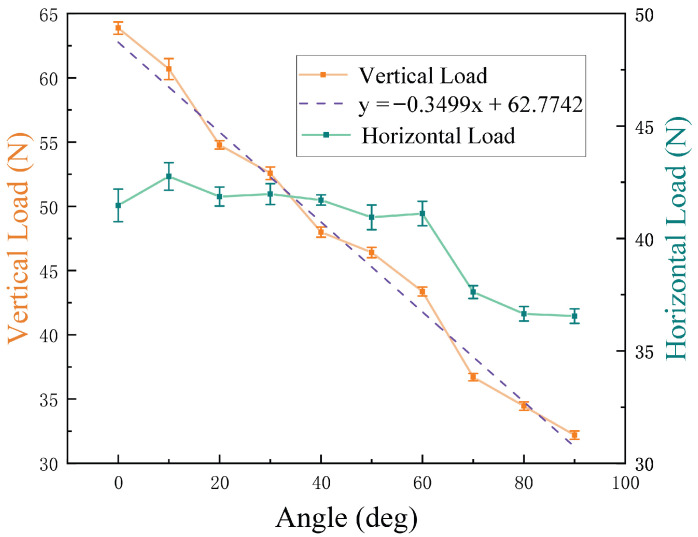
Plot of MC-1 loading capacity versus the inclined angle of the climbing plane.

From Figure 12, it can be found that as the tilt angle increases, the robot’s loading capacity in the vertical direction decreases more obviously. The loading capacity and tilt angle exhibit an approximately linear relationship. The linear fit of the coefficient of determination (i.e., $R^{2}$) produces a value of 0.9864.

In contrast, the change in loading capacity in the horizontal direction is not obvious. The results demonstrate the fine loading capacities of the robot. Compared to a modular robot [21] and tank-like climbing robot [23], MC-1 has a greater load capacity.

### 4.3. Testing Result of Wall-Climbing Speed

**Figure 13:**
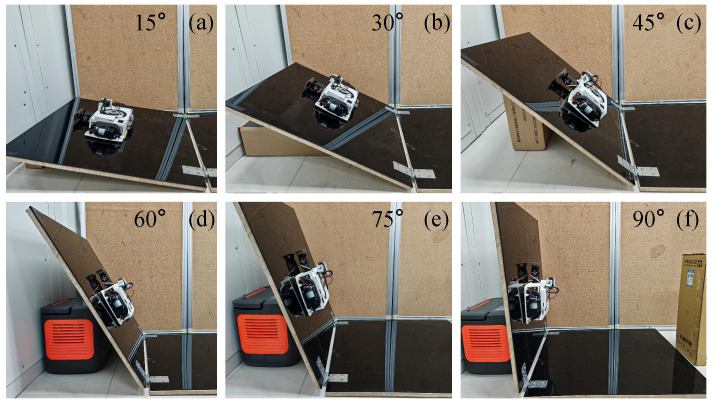
Movement speed test of MC-1 on the planes of different angles: (**a**) 15°; (**b**) 30°; (**c**) 45°; (**d**) 60°; (**e**) 75°; (**f**) 90°.

**Figure 14:**
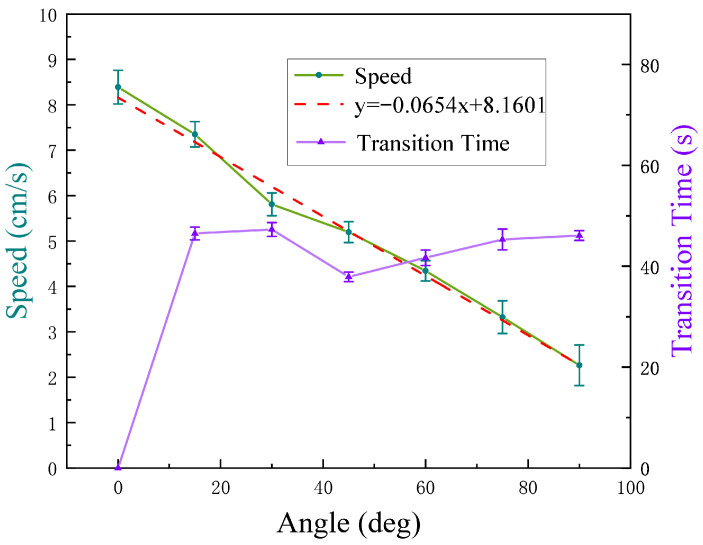
The wall-climbing speed and wall-transition speed versus the plane angle.

As shown in Figure 13, wall-climbing speeds directly affect the cleaning efficiency of the robot on the wall. In order to obtain the movement speed of the MC-1 robot on different inclined planes, seven groups of different angles in the interval of 0 to 90° were selected for testing. After the wall-climbing MC-1 robot’s module was stabilized, the time taken between its movement from the lowest point to the highest point on the wall was recorded. For the same inclined angle of the wall, five groups of data were measured repeatedly, and calculations were performed to obtain the mean and standard deviation of the movement speed of MC-1 in different angled planes, as recorded in Table 4. It is observed that the wall-climbing speed decreases from 8.39 cm/s to 2.27 cm/s as the inclination angle of the target plane increases from 0° to 90°. As depicted in Figure 14, the robot’s movement speed gradually decreases as the angle increases, and they exhibit a linear relationship. The coefficient of determination ($R^{2}$) for the linear fit has a value of 0.9843.

### 4.4. Testing Result of Plane-Transition Speed

**Figure 15:**
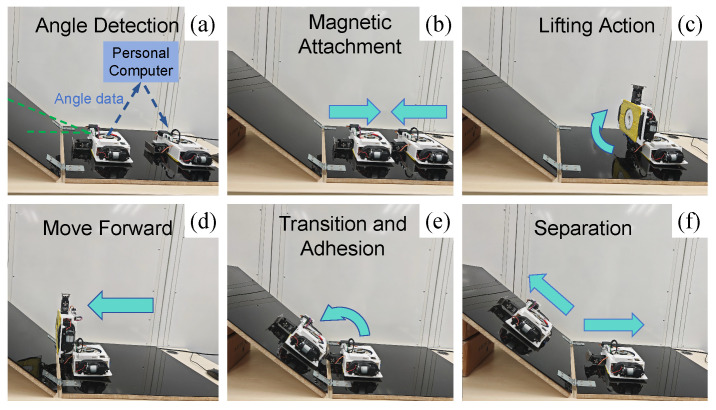
Transition time test of MC-1 on the planes of different angles. Main processes in wall-transition testing: (**a**) angle detection; (**b**) magnetic attachment; (**c**) lifting action; (**d**) move forward; (**e**) transition and adhesion; (**f**) separation.

Another crucial characteristic of facade-cleaning robots is their agility [5]. The plane-transition speed is one of the important indices for measuring the efficiency of the modular robot’s movement. A test of plane-transition speed can clearly demonstrate the coordination performance among various mechanisms and modules of the MC-1 robot. As shown in Figure 15, the experiment is conducted to record the total time it takes for the two modules of the MC-1 robot to complete a series of actions, including detecting angles, magnetic attachment, lifting and moving actions, adhesion, and the separation of two modules. The experiment tested the plane-transition times for a total of six different sets of angles from 15 to 90°, and the data are summarized in Table 5. The wall-transition time for each angle was measured five times, and the mean, standard deviation, and coefficient of variation of the transition time can be obtained. It is found that the coefficient of variation is not greater than 5%.

As shown in Figure 14, the time needed for the MC-1 robot to perform a wall transition does not change much as the angle increases, and the time is around 40 s. The experimental results show that the MC-1 modular cleaning robot has the ability to perform stable multi-plane cleaning, along with its capabilities to carry out object-bearing climbing (maximum load of 32.19 N), multi-angle plane measuring (angles between 0 and 90°), and cleaning.

Table 6 summarizes the performance of the robot’s work in comparison to several typical modular wall-climbing robots. The values of MC-1’s traveling speed and loading capacity in Table 6 are taken from the case of the 90° wall motion. Negative-pressure adhesion allows the MC-1 robot to adapt to a wide range of material planes compared to magnetically adsorbed robots. It can withstand a maximum of more than twice its own pendant load on a 90° plane. The combination of ultrasonic sensors and servo motors helps the robot to measure the angle of a plane, which broadens the application range of the MC-1 robot. Moreover, magnetic attachment between the MC-1 modules has the advantage of being reliable, and attachment and detachment occur quickly.

## 5. Conclusions

This paper presents a new design of the MC-1 robot for wall cleaning. The robot features a multi-module design for transition between multiple angles and utilizes a centrifugal fan to create a negative-pressure zone, ensuring stable surface adhesion. Additionally, the integration of a servo motor and ultrasonic sensors enables the robot to perform angular measurements of wall surfaces using integrated and cost-effective components. Conventional cleaning robots are limited to cleaning tasks on a single plane. In contrast, MC-1’s multiple modules can seamlessly transition between different wall surfaces. The theoretical analysis and experimental results demonstrate that MC-1 can reliably transition between multiple planes with varying inclinations. By leveraging the ultrasonic module to accurately measure the angle between the target and current planes and enabling multiple modules to work concurrently, the robot significantly enhances cleaning efficiency. In the future, the robot’s development will focus on improving environmental awareness and enabling autonomous path planning, further reducing human intervention and the workload of operation personnel. 

## Figures and Tables

**Table 1:** Main dimensions of MC-1 module.

*l*	$l_{1}$	$l_{2}$	$l_{3}$	$l_{4}$	*h*
221 mm	141 mm	110 mm	80 mm	111 mm	73.5 mm
$h_{c}$	*b*	$b_{s}$	$b_{t}$	*d*	$h_{p}$
27.1 mm	280 mm	234.4 mm	74 mm	20 mm	20 mm

**Table 2:** Suction force test results of the MC-1 module.

Angle (°)	Mean (N)	Standard Deviation	Maximum (N)	Minimum (N)
0	37.01	0.37	37.63	36.47
90	34.11	0.28	34.52	33.65

**Table 3:** Loading results of MC-1 on planes with different angles.

Angle (°)	0	10	20	30	40
Vertical (N)	63.88±0.48	60.69±0.81	54.78±0.32	52.58±0.49	47.99±0.40
Horizontal (N)	41.47±0.72	42.76±0.62	41.86±0.42	41.97±0.46	41.71±0.23
Angle (°)	50	60	70	80	90
Vertical (N)	46.41±0.41	43.37±0.35	36.71±0.29	34.46±0.32	32.19±0.32
Horizontal (N)	40.94±0.55	41.11±0.54	37.62±0.29	36.65±0.32	36.55±0.32

**Table 4:** Speed results of MC-1 on planes of different angles.

Angle (°)	0	15	30	45
Speed (cm/s)	8.39±0.37	7.35±0.28	5.81±0.25	5.20±0.23
Angle (°)	60	75	90	–
Speed (cm/s)	4.34±0.22	3.32±0.36	2.27±0.24	–

**Table 5:** Test results of the MC-1 plane-transition times at different angles.

Angle (°)	0	15	30	45	60	75	90
Transition time (s)	0	46.5±1.3	47.3±1.4	37.9±0.9	41.7±1.5	45.3±2.1	46.1±0.9
Coefficient of Variation (%)	0	2.8	3.0	2.4	3.6	4.6	2.0

**Table 6:** Performance comparison of typical modular climbing robots.

Specifications	This Work (MC-1)	R-Track [15]	Modular Robot [21]	Mantis [22]	Tank-Like Robot [23]	ROMERIN [24]
Adhesion	Negative pressure	Magnetic adhesion	Negative pressure	Negative pressure	Flat elastomer	Negative pressure
Locomotion	Rubber track	Magnetic track	Legged	Rubber track	Rubber track	Legged
Mass (g)	1350	2750	47	8000	180	2026
Modular	Yes	Yes	Yes	Yes	Yes	Yes
Wall-transition Speed (cm/s)	2.020	–	0.056	–	6	–
Loading (N)	32.24	–	15	–	5	77.4
Plane types	Smooth planes	Magnetism surfaces	Smooth planes	Smooth planes	Dry surfaces	Smooth planes

## Data Availability

All data are available in the main text.
